# Nonsensical choices? Fall armyworm moths choose seemingly best or worst hosts for their larvae, but neonate larvae make their own choices

**DOI:** 10.1371/journal.pone.0197628

**Published:** 2018-05-24

**Authors:** Julio C. Rojas, Michael V. Kolomiets, Julio S. Bernal

**Affiliations:** 1 Department of Entomology, Texas A&M University, College Station, TX, United States of America; 2 Department of Plant Pathology and Microbiology, Texas A&M University, College Station, TX, United States of America; University of Tennessee, UNITED STATES

## Abstract

Selecting optimal host plants is critical for herbivorous insects, such as fall armyworm (*Spodoptera frugiperda*), an important maize pest in the Americas and Africa. Fall armyworm larvae are presumed to have limited mobility, hence female moths are presumed to be largely responsible for selecting hosts. We addressed host selection by fall armyworm moths and neonate and older (3^rd^-instar) larvae, as mediated by resistance and herbivory in maize plants. Thus, we compared discrimination among three maize cultivars with varying degrees of resistance to fall armyworm, and between plants subjected or not to two types of herbivory. The cultivars were: (i) susceptible, and deficient in jasmonic acid (JA) production and green leaf volatiles (GLV) emissions (inbred line B73-*lox10*); (ii) modestly resistant (B73), and; (iii) highly resistant (Mp708). The herbivory types were: (i) ongoing (= fall armyworm larvae present), and; (ii) future (= fall armyworm eggs present). In choice tests, moths laid more eggs on the highly resistant cultivar, and least on the susceptible cultivar, though on those cultivars larvae performed poorest and best, respectively. In the context of herbivory, moths laid more eggs: (i) on plants subject to versus free of future herbivory, regardless of whether plants were deficient or not in JA and GLV production; (ii) on plants subject versus free of ongoing herbivory, and; (iii) on plants not deficient in compared to deficient in JA and GLV production. Neonate larvae dispersed aerially from host plants (i.e. ballooned), and most larvae colonized the modestly resistant cultivar, and fewest the highly resistant cultivar, suggesting quasi-directional, directed aerial descent. Finally, dispersing older larvae did not discriminate among the three maize cultivars, nor between maize plants and (plastic) model maize plants, suggesting random, visually-oriented dispersal. Our results were used to assemble a model of host selection by fall armyworm moths and larvae, including recommendations for future research.

## Introduction

Host plant selection by female herbivorous insects fundamentally affects their offspring’s survival and reproduction, and is particularly relevant to neonate offspring because they are especially susceptible to plant defenses, and must cope with a variety of obstacles to successfully colonize a host [1,2]. In the process of selecting host plants, female insects must discriminate among hosts at two levels, at least. First, they must discriminate among host and non-host plants, and second, they must discriminate among host plants of different qualities [2–4]. Discriminating among host and non-host plants typically entails discrimination among a discrete number of plant species, depending on habitat complexity (e.g., natural vs. agricultural habitats); discriminating among hosts entails discrimination among plants of varying quality, itself dependent on the interplay among a large number of frequently interacting plant (e.g., genotype, nutritional state, etc.) and environmental (e.g., drought stress, herbivory, etc.) variables. For example, a relevant host plant variable is nutritional state, which may vary quantitatively and qualitatively with age and tissue within plant individuals, and with environment and genotype across plant populations. Thus, cotton plants (*Gossypium hirsutum* L.) subjected to enhanced nitrogen fertilization were preferred by *Spodoptera exigua* (Hübner) females and larvae for oviposition and feeding, respectively [5]. An environmental variable relevant to host selection is herbivory, which may be differently important depending on its timing, whether prior, present or future, and whether it induces defensive responses in host plants. For instance, prior herbivory may affect host quality by mediating the amount of food available to a female’s offspring, while present and future herbivory may do so by mediating plant chemical responses to herbivory. Accordingly, previous studies showed that ovipositing females may discriminate against host plants with reduced biomass or in favor of plants with increased biomass due to prior herbivory [6,7]. Other studies showed that present and future herbivory, such as by insect larvae or eggs, respectively, may affect host selection by mediating the deployment of inducible direct and indirect chemical defenses against an herbivore’s offspring (e.g., [8–10]).

Discrimination among host plant individuals (rather than host plant species) is likely the prevailing situation in agricultural landscapes. There, single crops (i.e. host plants) may be dominant, though a range of crop cultivars may coexist across landscapes. Such is the context in which insect herbivores of maize (*Zea mays mays* L.) may search for hosts in areas of subtropical and tropical America and sub-Saharan Africa where the crop is grown by smallholder traditional farmers. In those areas, maize may be grown in landscapes dominated by mosaics of genetically-narrow, commercial hybrids or by assortments of genetically heterogeneous landraces. Moreover, in some areas of subtropical America, teosintes (species of *Zea* L. other than maize), the wild relatives of maize, may grow within fields of hybrid or landrace maize cultivars.

In the American tropics and subtropics, fall armyworm [*Spodoptera frugiperda* (J. E. Smith)] is typically the single-most important maize pest, particularly during the crop’s early, vegetative growth stages [11–16]. Importantly, it recently became a devastating, invasive maize pest in sub-Saharan Africa [17,18]. Fall armyworm is polyphagous, and known from >180 host plants from several families, though species of Poaceae (e.g., maize, sorghum, rice) are preferred [11,19–23]. In its larval stage, it can completely defoliate seedling and early-vegetative stage maize plants, stunt plant growth, or kill seedlings [11,12, 14,17,24]. A modest amount of research addressed the ecology of oviposition and larval dispersal on maize in the contexts of late-vegetative to reproductive stage plants, as well as within-plant movement of larvae (e.g., [25–31]). In contrast, however, comparatively little research addressed those questions in the contexts of seedling stage maize and between-plant dispersal of larvae, despite the susceptibility of seedlings to herbivory, and the relevance of oviposition and larval dispersal to the overall ecology of fall armyworm in the field (e.g., [12,16,32,33]).

A considerable number of studies have reported that maize seedlings respond to herbivory, including by fall armyworm larvae, by altering their direct and indirect induced defenses (e.g., [34–39]). Induced direct defenses affect insect performance, while induced indirect defenses attract natural enemies [38,40–42]. Prior studies addressed host plant selection by fall armyworm in relation to their offspring’s performance on variably defended maize cultivars, i.e. the performance-preference hypothesis [2,43], though they provided conflicting results. For example, an early study showed clear ovipositional discrimination among maize cultivars known to vary in their resistance to fall armyworm larvae [27], while other studies showed indiscriminate oviposition on host and non-host plants and non-plant objects, and no correlation between ovipositional preference and larval performance [19, 20, 32–44]. Aside from ovipositional preference based on offspring performance, some studies addressed whether present herbivory mediates host selection by ovipositing females. In the case of fall armyworm, one study showed that females were less attracted to maize plants injured by conspecific larvae compared to uninjured plants [45], though whether actual oviposition was mediated by plant injury was not evaluated. Similarly, little is known concerning dispersal, and feeding (host) preferences of young fall armyworm larvae, which typically disperse from the natal host shortly after eclosion [11,20,24,30,46]. Generally, neonate larvae are believed to disperse randomly from plants because they engage in ballooning, i.e. a means of dispersal in which neonate larvae are carried by wind currents captured by strands of silk produced by labial silk glands [1,12]. While prior laboratory studies showed that fall armyworm larvae are differentially attracted to maize cultivars or grasses that vary in their defense levels or nutritional qualities [44,47], available studies have not addressed whether neonate larvae disperse randomly, nor whether larvae that initially colonize an unsuitable host will disperse further, either directionally or randomly.

Plants may respond to both present herbivory, e.g., due to feeding by insect larvae, and future herbivory, e.g., as represented by insect eggs, through direct or indirect induced defenses (e.g., [10,48–50]). Importantly, insect oviposition may suppress some plant defenses, and prime others [51,52]. For example, a recent study reported that oviposition on maize by fall armyworm suppressed both constitutive (linalool) and induced host plant volatiles (terpenes and aromatic compounds) relevant to host finding by parasitoids [53]. Overall, that study’s results suggested that oviposition by fall armyworm females rendered plants less apparent to larval parasitoids and other females, potentially making them more suitable hosts by reducing parasitism risks and intraspecific competition [53]. Interestingly, though, other studies showed that oviposition by the stemborer *Chilo partellus* (Swinhoe) induced some maize cultivars, but not others, to release plant volatiles attractive to parasitoids [54–56]. Seemingly, the responses of maize plants to insect oviposition depend on herbivore species as well as maize cultivar. Thus far, however, it is unknown whether future herbivory, in the form of prior, conspecific oviposition, mediates host selection by fall armyworm females.

The objective of this study was to broaden our knowledge of fall armyworm’s ecology, particularly of: (i) host selection by females in relation to variable host plant quality, and present and future herbivory, and; (ii) dispersal and host preferences of young and older larvae. We focused our study on adult females, neonate larvae, and older larvae because these life-stages play distinct roles in fall armyworm’s life cycle and ecology. In particular, here we addressed whether: (i) ovipositing females discriminate among maize genotypes that differ in their suitability for larvae; (ii) any discrimination among maize genotypes by females is mediated by herbivory and its timing, future (presence of conspecific eggs) or present (presence of conspecific larvae), as well as by emission of herbivory-induced green leaf volatiles, and; (iii) dispersing neonate and older larvae forage directionally and discriminate among maize genotypes. Our results are especially relevant to fall armyworm’s status as a pest of seedling and early-vegetative stage maize, as is commonly the case in tropical and subtropical America, and recently sub-Saharan Africa [14–18]. Combined with the results of prior studies, our results were used to (i) assemble a hypothetical model of oviposition and larval ecology, and point to knowledge gaps therein to be addressed in future research, and (ii) discuss management strategies for contexts in which fall armyworm is a pest of seedling and early-vegetative stage maize.

## Materials and methods

### Insects and plants

Fall armyworm eggs, larvae (3^rd^-instar), and pupae, as required for each experiment, were purchased from Benzon Research, Inc. (Carlisle, PA, USA). The fall armyworm culture originated from specimens collected in Mississippi, USA, and consists mostly of corn host race individuals (94%; rice host race = 6%) (pers. comm. Chad Finkenbinder, Benzon Research Carlisle, Pennsylvania). Three maize inbred lines, B73, Mp708, and B73-*lox10*, selected on the basis of known resistance or susceptibility to fall armyworm, were used in this study, unless noted otherwise. B73-*lox10* (*lox10-3* mutant allele) is a jasmonic acid- (JA), terpene-, and green leaf volatile (GLV)-deficient mutant line in B73 background (backcrossed seven times to B73, > 99% similarity to B73), and was included as a susceptible host, relative to B73 [57]; B73, is a reference inbred line, and was included as an intermediately resistant host, relative to B73-*lox10*, and Mp708 [57,58], and; Mp708 is an inbred line bred for resistance to fall armyworm, and was included as an overall resistant host [36,58–60]. B73-*lox10* and B73 seed were produced by MVK; Mp708 seed was obtained from USDA National Plant Germplasm System, North Central Regional PI Station, Ames, Iowa (Plant ID PI 536520). A fourth inbred line, W438, was used in one experiment, as described below, and seed was produced by MVK. Seedlings were grown in greenhouse soil (Metro-Mix 350 Growing Mix, Sun Gro Horticulture, Agawam, MA, USA) in plastic cone-tainers (20 cm high × 4 cm top diam × 1.5 cm bottom diam; Ray Leach Cone-tainer SC-10 https://www.stuewe.com/products/rayleach.php) under artificial lighting (12 light: 12 dark h), and temperature of 24–31°C. Three-week-old seedlings (V4 or V5 stage) were used in all experiments.

### Adult host preference *vis-à-vis* offspring performance

#### Larval performance

This experiment addressed whether fall armyworm performance was mediated by feeding on any of three inbred lines, B73, B73-*lox10*, and Mp708. We used growth (weight gain) over 4 d as a proxy for performance in order to minimize larval mortality during molting and attributable to inbred line Mp708 over longer periods [36,59,60]. The experiment consisted in placing a 10-day old (3^rd^ instar) larva onto a plant of each inbred line in a cone-tainer. Larva-infested plants were maintained in a climate-controlled room (12 light: 12 dark h, 24–27°C, 50–70% R.H.). Each plant was enclosed within a 2.0 L PET bottle (placed inverted on cone-tainer; mouth opening removed to snugly fit around cone-tainer, and bottom removed and covered with fine mesh to provide ventilation) to prevent escape of larva. Each larva was weighed before its placement on a plant (day 0), and was recovered and weighed 4 d later. All larvae were killed by freezing, and then dried to constant weight in an oven at 80°C for ≥3 d. Twenty-five larvae were assayed per each inbred line. We expected that performance of fall armyworm larvae would be best on B73-*lox10* inbred line, followed by B73 and Mp708, per previous studies [57,58].

Statistical analyses consisted of analyses of covariance (ANCOVA) of larvae dry weights after 4 d, including plant type (i.e., B73, B73-*lox10*, Mp708) as independent variable, and larva fresh weight at the beginning of the assay as independent covariable. Larvae weights were normalized by converting them to their natural log values prior to ANCOVA. Tukey’s post-hoc tests were used as warranted for comparing among means of larva dry weight. Results are presented as back-transformed means. All analyses were conducted using JMP Pro 13.1.0 [61].

#### Adult host preference

This experiment addressed whether fall armyworm females discriminate among maize seedlings that differently affect their offspring’s performance, as measured in the experiment assessing larval performance (see above). This experiment included the three maize inbred lines evaluated in the larval performance experiment, B73-*lox10*, B73, and Mp708. We hypothesized that the ovipositional preferences of fall armyworm females for the maize inbred lines would reflect the performance of their offspring on those inbred lines.

A total of 31 independent trials were conducted, each within a collapsible mesh cage (90 cm × 76 cm × 76 cm, Live Monarch Foundation, Boca Raton, FL, USA). In each trial, three plants (one per maize inbred line), each growing in a cone-tainer, were placed inside the mesh cage; each plant was maintained upright by burying the cone-tainer in a pot filled with soil. The three plants were positioned in a triangular formation, separated one plant from another by ~30 cm; plant position was independently randomized in each trial. Ten pupae (5 females and 5 males) were placed in a Petri dish at the cage center 2 d before emergence of moths was expected; a 10% sucrose solution, dispensed in cotton wool, was also placed at the cage center as food for emerging moths. After moth emergence, plants were checked twice daily for presence of fall armyworm egg masses; a trial was terminated when the first eggs were found. We found egg masses on cage sides in 14 cases, but in every case the number of masses on plants exceeded the number on cage sides. The numbers of egg masses and eggs per plant, and plant height (measured from the base of the stem to tip of the youngest leaf) were recorded per each trial. The photoperiod cycle used in the room where the trials were conducted was 12: 12 h, with the dark phase beginning at 19:00 h, and the ambient temperature was 21–23°C.

Statistical analyses consisted of ANCOVA of rank-transformed numbers of eggs and egg masses found on seedlings after 24h, including plant type (i.e., B73, B73-*lox10*, Mp708) as independent variable, and seedling height as covariable. Tukey’s post-hoc tests were used as warranted for comparing among the mean rank numbers of eggs or egg masses on the different plant types. All analyses were conducted using JMP Pro 13.1.0 [61], and results are presented as means and errors of original data.

### Adult host preference *vis-à-vis* herbivory

#### Future herbivory

This experiment addressed whether fall armyworm females discriminate between maize seedlings bearing conspecific eggs and seedlings free of eggs, and whether any discrimination is mediated by emission of green leaf volatiles (GLV) and terpenes. Therefore, the experiment included two independent variables, (i) presence (or absence) of fall armyworm eggs, and (ii) presence (or absence) of GLVs and terpenes. Two maize inbred lines were used to evaluate the second variable: B73, which produces GLVs and terpenes, and B73-*lox10*, which does not. We hypothesized that fall armyworm females would discriminate against B73 seedlings with conspecific eggs, but not against B73-*lox10* seedlings with eggs, because these seedlings do not emit GLVs and terpenes [57].

Seedlings bearing fall armyworm eggs were obtained by releasing at ~8:00h 10 mated females (3-5-d old) inside a collapsible cage (61 × 34 × 34 cm, Live Monarch Foundation, Boca Raton, Fl, USA) containing 6–8 seedlings of a single inbred line (B73 or B73-*lox10*). Seedlings were removed from the cage 24h later (after the dark phase, which extended from 19:00 to 07:00 h), and examined for egg masses; any egg masses found were marked with a surrounding circle (Sharpie® Ultra Fine Marker Black, http://www.sharpie.com), and the numbers of egg masses and eggs on seedlings were recorded at this time (on average, treated B73 seedlings bore 163 ± 21 eggs in 1.6 ± 0.1 masses, while treated B73-*lox10* seedlings bore 126 ± 20 eggs in 1.4 ± 0.1 masses). Seedlings bearing egg masses were set aside and used in the experiment within ~2 h; seedlings without egg masses used in the experiment were not previously exposed to fall armyworm females.

A total of 25 trials per each inbred line were conducted within collapsible mesh cages (90 cm × 75 cm × 75 cm). For each trial, two seedlings of one inbred line, one with- the other without egg masses, were placed at opposite corners within each cage, ~50 cm distant from each other; seedling position was randomly assigned per each trial. Five mated females were introduced in each cage at ~10:00h; 10% sugar-water solution, dispensed in soaked cotton wool, was placed in the center of cage as food for adults. Seedlings were examined 24h later for egg masses, and the numbers of masses and eggs per seedling were recorded. We found egg masses on cage sides in nine cases, but in every case the number of masses on plants exceeded the number on cage sides. The photoperiod cycle used in the room where the cages were placed was 12: 12 h, with the dark phase beginning at 19:00h, and temperature at 21–23°C.

Statistical analyses consisted of ANOVA of rank-transformed numbers of eggs and egg masses on seedlings after 24h, including plant type (i.e., B73, B73-*lox10*), prior oviposition, and their interaction as independent variables. Prior assays (see *Adult host preference*) showed that seedling height did not significantly mediate host preference (P = 0.300–0.816); consequently, and to minimize injury to plants due to handling, this variable was not measured nor included in the statistical analysis. Moreover, exploratory analyses showed that neither the number of eggs or egg masses laid on seedlings subjected to prior oviposition mediated host preference (P = 0.467–0.827), so initial egg and egg mass numbers were not included as covariables. Tukey’s post-hoc tests were used as warranted for comparing among the mean rank numbers of eggs or egg masses on the different plant types; results are presented as means and errors of original data. All analyses were conducted using JMP Pro 13.1.0 [61].

#### Present herbivory

This experiment addressed whether fall armyworm females discriminate between maize seedlings with conspecific larvae (present herbivory) or without larvae, and whether any discrimination was mediated by emission of GLVs and terpenes. Therefore, the experiment included two independent variables, (i) presence (or absence) of fall armyworm larvae and injury, and (ii) presence (or absence) of GLVs and terpenes. Inbred lines B73, which produces GLVs and terpenes, and B73-*lox10*, which does not, were used to evaluate the second variable. Similar to our expectations in the future herbivory experiment, we expected that fall armyworm females would discriminate against B73 seedlings injured by conspecific larvae, but not against B73-*lox10* seedlings damaged by larvae, because the latter seedlings were deficient in JA production and GLV and terpene emissions [57].

Seedlings with fall armyworm injury were obtained by caging three 3^rd^-instar larvae in a clip-cage on treated seedlings in each trial (preliminary assays showed that similar amounts of injury were produced on B73 and B73-*lox10* seedlings by using three 3^rd^-instar larvae); untreated seedlings received clip-cages, but not larvae. The clip-cages had a 3.5 cm inner diameter (area ≈ 9.6 cm^2^), and larvae were allowed to feed for 3 h beginning 7–8 h prior to a trial. A total of 22 independent trials were conducted similar to those described for future herbivory, with modifications pertinent to evaluating the effects of present herbivory. Thus, seedlings exposed to fall armyworm larvae inside clip cages or clip cages alone replaced seedlings bearing or not bearing fall armyworm eggs. As in the trials addressing the effects of future herbivory, fall armyworm females in this experiment were allowed to oviposit for 24 h, after which seedlings were removed from cages to record the numbers of egg masses and eggs found on each seedling. We found egg masses on cage sides in eight cases, but in every case the number of masses on plants exceeded the number on cage sides.

Statistical analyses consisted ANOVA of rank-transformed numbers of eggs and egg masses found on seedlings after 24h, including plant type (i.e., B73, B73-*lox10*), prior injury, and their interaction as independent variables. Prior assays (see *Adult host preference*) showed that seedling height did not significantly mediate host preference, so this parameter was not included as a covariable. Results are presented as means and errors of original data (eggs and egg masses per seedling). All analyses were conducted using JMP Pro 13.1.0 [61].

### Host finding and preference of offspring

#### Host finding and preference of neonate larvae

This experiment addressed whether following their eclosion from eggs fall armyworm larvae disperse, colonize and settle on host plants at random or according to variable resistance and GLV and terpene emissions in seedlings. Thus, over the course of 4.5 d we recorded the numbers of neonate larvae on seedlings of B73, B73-*lox10*, and Mp708 after having dispersed from their natal host seedling (maize inbred line W438). We hypothesized that because neonate larvae disperse non-directionally, as mediated by prevailing winds (i.e., ballooning), they would randomly colonize the available seedlings, but following colonization may disperse in search of a seedling more suitable than the colonized seedling.

A total of 9 trials were conducted over three dates, with three trials on each date. In each trial fall armyworm eggs were placed on a seedling (inbred line W438) located at the center of a circular arena. The eggs were allowed to hatch, and the numbers of larvae on each of six seedlings (two per inbred line, and deployed equidistantly along the arena’s perimeter) were recorded. The arena consisted of a circular wading pool (152.4 cm diam × 15.2 cm depth) (General Foam Plastics Corp., Norfolk, Va. 23502) filled to ~14 cm deep with soil. A seedling (W438) grown in 9 × 10 × 10 cm pot was buried at the arena’s center so that the pot’s upper edges were ~1cm below the soil level. A paper-clip was used to attach an egg mass (~200 eggs, on sections of paper) at dusk (~20:00h) to the center seedling’s whorl leaf; egg masses were deployed when the eggs had darkened, indicating that they were proximate to hatching. Prior observations and preliminary trials showed that, with few exceptions, all darkened eggs hatched within 24 h, and all larvae dispersed from the center seedling (bearing the egg mass), which was “skeletonized,” within 4 d. Ballooning by neonate larvae was (observed and) facilitated by wind currents (0.09 ± 0.01–0.25 ± 0.01 m/sec at arena center) created by the greenhouse’s fanning system, though arenas were protected from direct wind currents on four sides by wooden barriers (61 cm-high, 183 cm-long, 1.3 cm-thick plywood). Six seedlings, i.e. two per each inbred line (B73, B73-*lox10*, Mp708), were buried along the arena’s perimeter at nominal positions 2, 4, 6, 8, 10, and 12, which were separated by ~59 cm from each other; the distance from each seedling to the seedling with eggs at the arena’s center was ~56 cm; positions 12 and 6 were fixed at the arena’s south and north ends, respectively. The size (total length of main stem and leaves) of each seedling was measured so that only similarly-sized seedlings (~2 cm difference between largest and smallest seedling) were included in each trial, and each seedling’s position in the arena was assigned randomly in each trial. The numbers of larvae per each seedling were counted beginning ~20 h after deploying the eggs and over the following 4.5 d at ~8:00 h and 20:00 h.

Statistical analyses included the per-arena (two seedlings) per plant type cumulative frequencies of larvae recorded during the initial (0–24 h) and final (84–108 h) 24 h periods of the 4.5 d trial, each period indicating different stages in larval foraging. The observations recorded during the initial 24 h period were considered a measure of colonization by ballooning, neonate larvae; those of the final 24 h were considered a measure of settling by larvae. Statistical analysis consisted of repeated-measures ANOVA of rank-transformed, cumulative larval frequencies, with period (colonization, 0–24 h; settling, 84–108 h) as repeated measure, plant type (B73, B73-*lox10*, Mp708) as independent variable. Tukey’s post-hoc tests were used as warranted for comparing among means; results are presented as means of cumulative frequencies of larvae and corresponding standard errors. All analyses were conducted using JMP Pro 13.1.0 [61].

#### Host finding and preference of “older” larvae in relation to performance

This experiment addressed whether “older” (3^rd^-instar) fall armyworm larvae disperse and select host plants randomly, independently of variable resistance levels and their potential performance on different host plants. Thus, this experiment included the three maize inbred lines evaluated in the larval performance experiment, B73-*lox10*, B73, and Mp708. We hypothesized that larvae would display directional movement toward and settle on seedlings on which their performance is best, per the results of the experiment assessing larval performance (see above).

The preferences of larvae for the tested maize plants were evaluated in greenhouse choice tests in which three different seedlings were exposed to larvae. The test arena consisted of a circular tub (54 cm diam. × 41 cm high) (Mainstays, Walmart Stores Inc., Bentonville, AR) filled with soil to ~23 cm. One seedling of each inbred line growing in cone-tainers was buried in the soil to ~1cm above the cone-tainer’s mouth; the seedlings were positioned ~5cm from the tub’s perimeter and separated from each other by ~15 cm. Ten 3^rd^-instar larvae (previously starved for 2 h) were released on a filter paper (10 mm diameter) placed in the arena’s center. The arena´s inner wall was coated with Insect-a-Slip (BioQuip, Rancho Dominguez, CA) to prevent larvae from escaping the arena. The number of larvae on each of the seedlings was recorded at 2, 15, 60, 120, 180, 240, and 300 min. In total, 25 replicate trials were performed in this experiment.

Statistical analyses consisted of repeated measures analyses of variance (ANOVA) of rank-transformed numbers of larvae found on seedlings at each of the observation times; plant type (i.e., B73, B73-*lox10*, Mp708) was included as an independent variable, and the numbers of larvae on seedlings at each of the observation times as a repeated measure. Tukey’s post-hoc tests were used as warranted for comparing among the mean rank numbers of larvae on the different plant types. All analyses were conducted using JMP Pro 13.1.0 [61].

#### Host finding by “older” larvae in relation to host volatiles

This experiment addressed whether “older” (3^rd^-instar) fall armyworm larvae disperse randomly when searching for a host plant or rely on volatile cues for directed orientation at a distance. Thus, we compared the attraction of larvae to model seedlings (made of plastic and steel wire; Green Plastic Monkey Grass, trimmed to simulate a maize seedling; Hobby Lobby Stores, Inc., Oklahoma City, OK 73179) against maize seedlings of two types in independent experiments. In one experiment, we compared attraction to B73 maize seedlings against model seedlings, and in another experiment, we compared B73-*lox10* seedlings against model seedlings; B73-*lox10* seedlings are deficient in JA production and GLV and terpene emissions, as previously noted. We hypothesized that larvae would discriminate between B73 seedlings and model seedlings, but not between model seedlings and B73-*lox10* seedlings. Trials were independent, paired choice-tests for each maize seedling type, i.e. B73 seedling vs. model seedling, and B73-*lox10* seedling vs. model seedling, and were conducted in the arena described above (*Foraging by 3*^*rd*^*-instar larvae*). In these trials, however, three real seedlings and three model seedlings were placed in the arena. The seedlings of both types were placed alternately, ~8 cm from each other, and the arena was rotated in a clockwise direction after each trial. Ten 3^rd^-instar larvae (starved 2 h prior to their use) were released in the center of arena as described above. The number of larvae that contacted the seedlings was recorded in a 5 min period; larvae were removed from the arena immediately upon contacting a seedling. In total, nine replicate trials (i.e., 90 larvae total) were performed during this experiment.

Statistical analyses consisted of ANOVA of the rank-transformed numbers of times a seedling stem was encountered by a larva, and included plant type (i.e., B73 or B73-*lox10* maize inbred line), seedling nature (i.e., real or model seedling), and their interaction as independent variables. All analyses were conducted using JMP Pro 13.1.0 [61].

## Results

### Adult host preference *vis-à-vis* offspring performance

#### Larval performance

**Fig 1:**
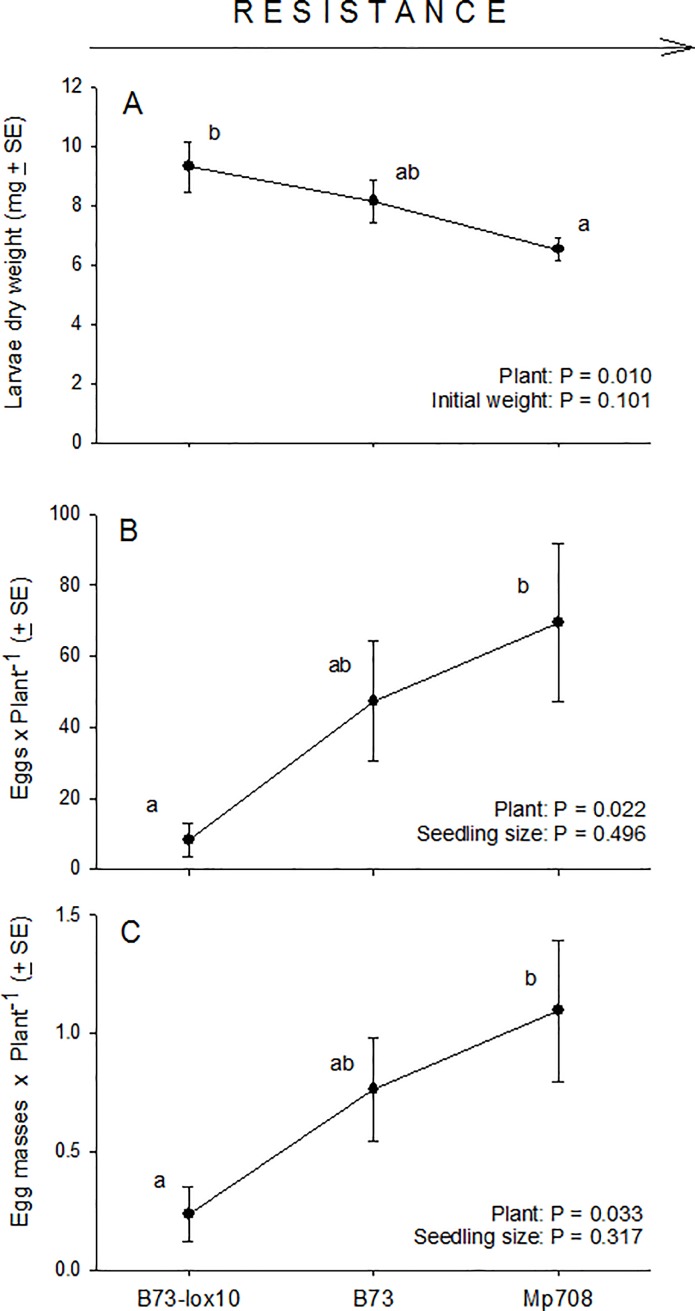
Performance of fall armyworm larvae on and ovipositional preferences of fall armyworm females for three increasingly resistant maize inbred lines, B73-*lox10*, B73, and Mp708. Performance of larvae was assessed as body dry weight (mg ± SE) after feeding for 4 d on seedlings of one maize inbred line **(A)**. Ovipositional preferences of fall armyworm females were assessed in choice-assays involving all three maize inbred lines, and measured as total eggs laid per individual seedling **(B)**, and total egg masses per each seedling **(C)**. Different lower-case letters above means within each plot indicate significant differences per ANOVA (critical P = 0.05) and Tukey’s tests. The maize inbred lines are ordered from left to right according to the presumed strength of their resistance to fall armyworm, as indicated at the top of the figure.

Larval dry weight after 4 d was mediated by plant type (F_2, 74_ = 4.95, P = 0.010), but not by initial larval weight (F_1, 74_ = 2.77, P = 0.101) (Fig 1A). Dry weight was greatest on B73-*lox10* seedlings, followed by B73 and Mp708 seedlings. These results were consistent with our expectation that the performance of 3^rd^-instar fall armyworm larvae would be poorest on the most resistant seedlings, Mp708 maize, followed by the moderately resistant B73 seedlings, and the susceptible B73-*lox10* seedlings.

#### Adult host preference

Oviposition by adult females was mediated by plant type, but not by seedling size (Fig 1B and 1C): Both the numbers of eggs per seedling (F_2, 62_ = 4.07, P = 0.022) (Fig 1B), and egg masses per seedling (F_2, 62_ = 3.62, P = 0.033) (Fig 1C) were mediated by plant type, though not by seedling height (F_1, 62_ ≤ 0.47, P ≥ 0.496). These results indicated that fall armyworm females discriminated among plant types, and suggested that, contrary to our expectations, their preferences were negatively correlated with the performance of 3^rd^-instar larvae on the different plant types (cf. Fig 1A). Indeed, correlation analysis showed that the mean number of eggs oviposited per plant type (this experiment) and corresponding mean larval dry weight (*Larval performance* experiment) were strongly and negatively correlated (Pearson’s r = 1.000, P = 0.005).

### Adult host preference vis-à-vis herbivory

#### Future herbivory

**Fig 2:**
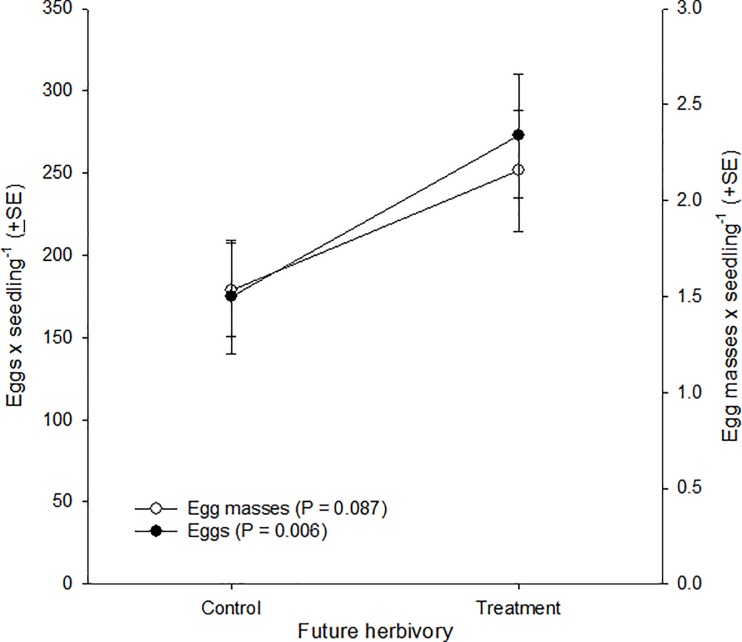
Ovipositional preference of fall armyworm females for maize seedlings subject to future herbivory (= hosting newly-laid, conspecific eggs) or seedlings exempt from future herbivory (= plants free of eggs) on two maize inbred lines, B73 and B73-*lox10*. Preference was assessed in a choice-test over a 24-h period, independently on each maize inbred line, and was measured as the numbers of eggs (filled circles) and egg masses (empty circles) laid by females on seedlings without (= Control) or with (= Treatment) eggs, which had been laid within 24–26 h prior to an assay. Overall, the numbers of eggs and egg masses laid per seedling were not mediated by maize inbred line (B73 or B73-*lox10*) (hence, circles represent means of both inbred lines) or by an interaction between maize inbred line and herbivory (future herbivory or no future herbivory) (F_1, 86_ ≤ 1.97, P ≥ 0.16).

Oviposition by adult females was mediated by prior oviposition (eggs: F_1,86_ = 8.015, P = 0.006; egg masses: F_1,86_ = 2.984, P = 0.087), but not by plant type (F_1,86_ ≤ 0.755, P ≥ 0.387), and these variables did not significantly interact with each other (F_1,86_ ≤ 1.972, P ≥ 0.164) (Fig 2). Females laid more eggs on seedlings with prior oviposition compared to seedlings without prior oviposition (P = 0.006), and while numerically they laid more egg masses on seedlings with prior- compared to seedlings without prior oviposition, the difference was not significant (P = 0.087). These results were inconsistent with our expectations. First, opposite to our expectation, fall armyworm oviposition was greater on seedlings on which future herbivory was expected compared to seedlings on which it was not, and; second, discordant with our expectation, this difference was not mediated by maize plant type, i.e. was independent of production of JA, GLVs and terpenes by seedlings.

#### Present herbivory

**Fig 3:**
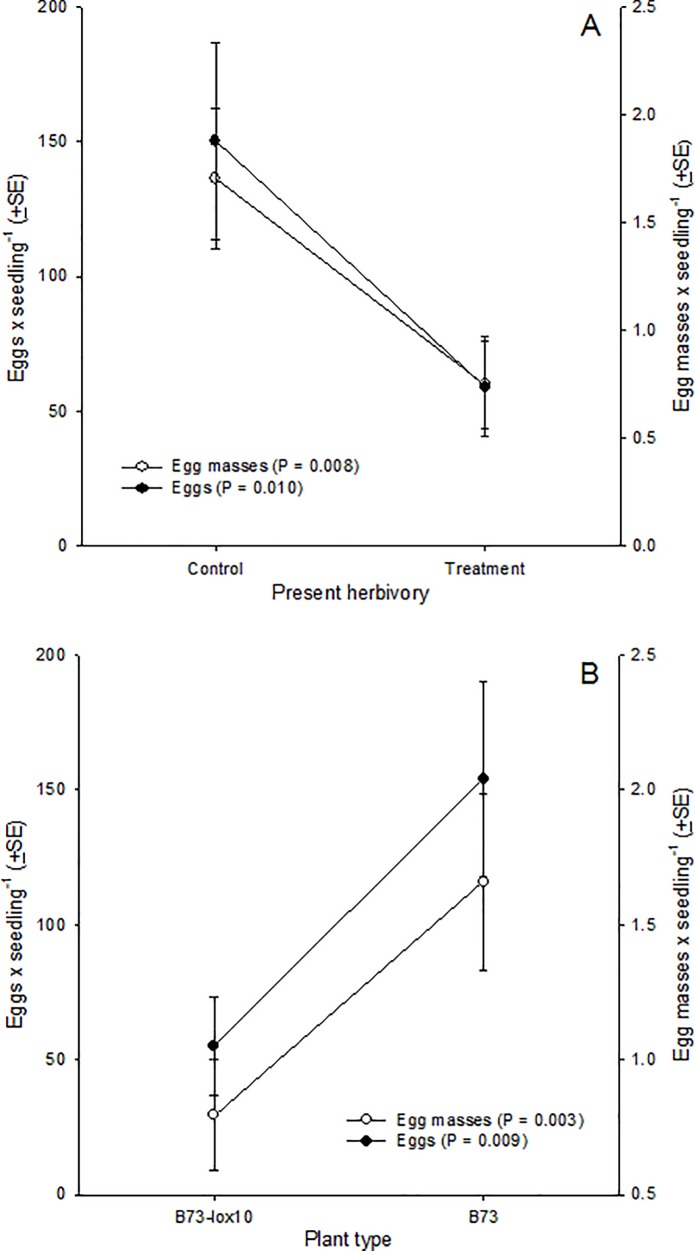
Ovipositional preference of fall armyworm females for maize plants subject to present herbivory (= exposed to conspecific larvae) or plants not subject to present herbivory (= plants not exposed to larvae) on two maize inbred lines, B73 and B73-*lox10*. Preference was assessed in a choice-test over a 24-h period, independently on each maize inbred line, and was measured as the numbers of eggs (filled circles) and egg masses (empty circles) laid by females on seedlings with (= Treatment) or without (= Control) feeding injury. Feeding injury was produced by confining three larvae on a seedling for 3 h, beginning 7–8 h prior to a trial. Overall, differences in eggs and egg masses laid per seedling were detected between seedlings with or without present herbivory **(A)**, and between B73 and B73-*lox10* maize inbred lines **(B)**, while a significant interaction between herbivory and maize inbred line was not found (F_1, 84_ ≤ 0.84, P ≥ 0.36).

Oviposition by adult females was mediated by prior injury and plant type (P ≤ 0.010), but not by any interaction between these variables (F_1,84_ ≤ 0.838, P ≥ 0.363) (Fig 3). Females laid fewer eggs (F_1,84_ = 6.936, P = 0.010) and egg masses (F_1,84_ = 7.451, P = 0.008) on seedlings with prior injury compared to healthy seedlings (Fig 3A). Similarly, females laid fewer eggs (F_1,84_ = 9.723, P = 0.003) and egg masses (F_1,84_ = 7.058, P = 0.009) on B73-*lox10* seedlings compared to B73 seedlings (Fig 3B). These results indicated that fall armyworm oviposition was lower on seedlings with present herbivory compared to seedlings without present herbivory, though discordant with our expectation the difference was not mediated by plant type; also, the results indicated that fewer eggs were laid on B73-*lox10* compared to B73 seedlings, independently of present herbivory.

### Host finding and preference of offspring

#### Host finding and preference of neonate larvae

**Fig 4:**
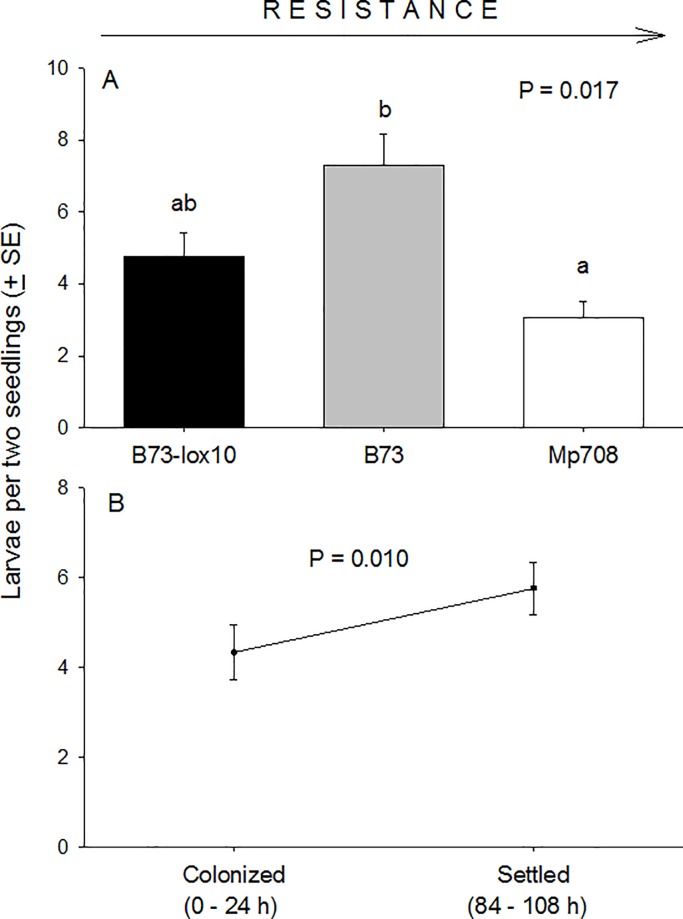
Colonization and settling on seedlings of three maize inbred lines, B73-*lox10*, B73, and Mp708 by neonate fall armyworm larvae. Two-hundred fall armyworm eggs were deployed at dusk on a seedling (maize inbred line W438) positioned at the center of a circular arena and surrounded with six seedlings (two of each: B73-*lox10*, B73, Mp708). The numbers of neonate larvae on each of the six seedlings (other than the seedling at the arena’s center) were counted at 0–24 h and 84–108 h after hatching; the larvae counted at 0–24 h were considered to have *colonized* a seedling, while those counted at 84–108 h were considered to have *settled* on a seedling. Differences in the frequency of colonization among seedlings of the three maize inbred lines **(A)**, and between colonizing and settled larvae **(B)** were detected, but no interaction was detected between maize inbred line and colonizing or settled larvae (F_2, 24_ = 0.610, P = 0.554). Different lower-case letters above means in **(A)** indicate significant differences per ANOVA (critical P = 0.05) and Tukey’s tests. The maize inbred lines in **(A)** are ordered from left to right according to the presumed strength of their host plant resistance to fall armyworm, as indicated at the top of the figure.

Host finding by neonate larvae was mediated by plant type (F_2, 24_ = 4.890, P = 0.017), and period (colonization and settling) (F_1, 24_ = 7.790, P = 0.010), though these variables acted independently of each other (F_2, 24_ = 0.610, P = 0.554) (Fig 4). Of larvae that colonized and settled on seedlings, ~48% did so on B73, ~32% on B73-*lox10*, and ~20% on Mp708 maize seedlings (Fig 4A), and slightly more larvae settled on seedlings (5.8 ± 0.6) compared to the number that initially colonized seedlings (4.3 ± 0.6) (Fig 4B). Notably, by 4.5 d after hatching only 2.9 ± 0.3 larvae were found on each of six seedlings within an arena, which amounts to 1.4% of the 200 eggs that were deployed per arena. These results were inconsistent with our expectations because they suggested that colonization of seedlings by neonate larvae was not random, and this was not affected by post-colonization dispersal.

#### Host finding and preference of “older” larvae in relation to performance

**Fig 5:**
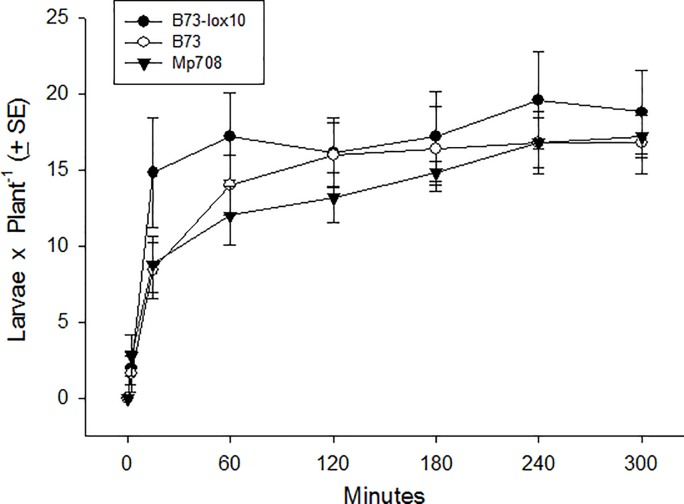
Host plant finding and preferences of older (3^rd^-instar) fall armyworm larvae for three maize inbred lines, B73-*lox10*, B73, and Mp708. Ten 3^rd^-instar larvae were released at the center of a circular arena in which one seedling of each of the three maize inbred lines were positioned near the arena’s margin, and the number of larvae on each of the seedlings was recorded after 2, 15, 60, 120, 180, 240, and 300 min. While increasingly more larvae were found on seedlings over time (F_6, 104_ = 35.41, P < 0.001), differences were not detected among the numbers of larvae on seedlings of the different maize inbred lines (F_2, 104_ = 0.56, P = 0.583), and the numbers of larvae on seedlings were unaffected by any interaction between maize inbred line and time (F_12, 104_ = 1.19, P = 0.309).

A small number of larvae found a maize seedling within 2–15 min, and most larvae found a seedling by 60 min (Time: F_6, 104_ = 35.41, P < 0.001) (Fig 5). However, significant differences were not detected among the overall numbers of larvae on the different plant types over the duration of the experiment (Plant type: F_2, 104_ = 0.56, P = 0.583), nor among the numbers of larvae on different plant types at any of the observation times (Plant type × Time: F_12, 104_ = 1.19, P = 0.309) (Fig 5). These results indicated that 3^rd^-instar fall armyworm larvae foraged randomly, and that upon encountering a seedling did not discriminate among plant types that differentially affect their performance. Thus, these results were contrary to our expectation that larvae would display directional movement toward and settle on seedlings on which their performance is best.

#### Host finding by “older” larvae in relation to host volatiles

**Fig 6:**
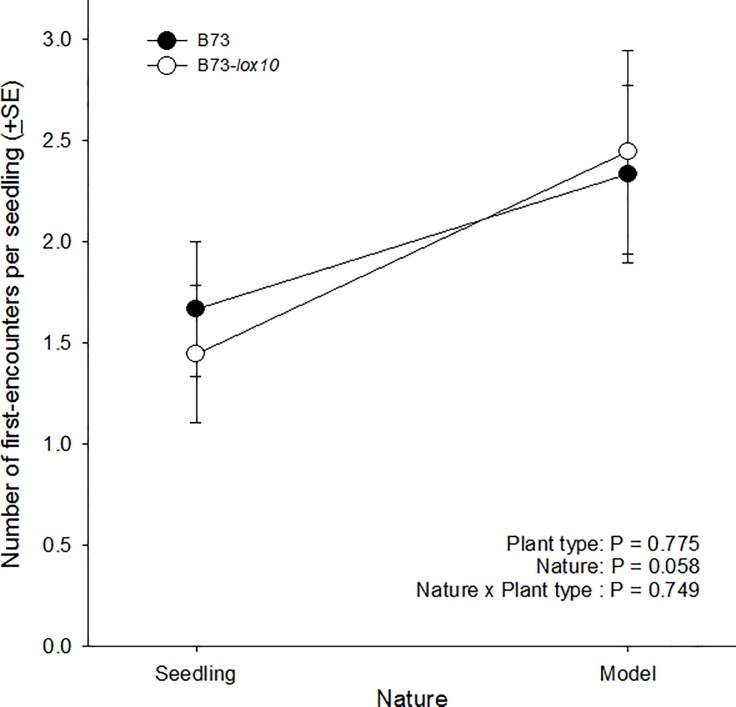
Host finding by older (3^rd^-instar) larvae in relation to host plant volatiles. The attraction of 3^rd^-instar larvae to model (constructed of plastic and metal wire) or real seedlings of two maize inbred lines, B73-*lox10* and B73, was assessed in a paired choice-test, independently for each inbred line. In each trial, ten 3^rd^-instar larvae were released in the center of a circular arena in which three each, model and real (B73 or B73-*lox10*) seedlings were positioned along the arena’s margin. After release, larvae were monitored for 5 min, and first-encounters of larvae with a seedling were recorded by observing then removing larvae immediately upon encountering a seedling. First-encounters of larvae with seedlings were unaffected by plant nature (model or real seedling, F_1, 32_ = 3.883, P = 0.058), maize inbred line (B73 or B73-*lox10*, F_1, 32_ = 0.083, P = 0.775), or any interaction between those variables (F_1, 32_ = 0.104, P = 0.749). In the plot, maize inbred line is indicated with open (B73-*lox10*) or closed (B73) circles, and seedling nature (real or model) is on the horizontal axis.

Most larvae crawled directly toward a seedling, whether real or model, while a minority of larvae crawled between seedlings until reaching the arena’s edge and changing course. However, the numbers of times a seedling of the different plant types was encountered first was independent of plant nature (F_1, 32_ = 3.883, P = 0.058), plant type (F_1, 32_ = 0.083, P = 0.775), and of any interaction between those variables (F_1, 32_ = 0.104, P = 0.749) (Fig 6). These results were inconsistent with our expectations because they indicated that 3^rd^-instar fall armyworm larvae foraged randomly, and suggested that volatile cues from seedlings were irrelevant to foraging larvae. Also, these results were inconsistent with our expectations that larvae would discriminate between JA-, GLV-, and terpene-producing seedlings (B73) and model seedlings, but not between model seedlings and JA and GLV-deficient seedlings (B73-*lox10*).

## Discussion

In this study, we investigated the host preferences and searching behavior of fall armyworm females and neonate and late-instar larvae, three ecologically relevant stages in this herbivore’s life cycle. Generally, our expectation was that within the limitations of their mobility both females and larvae would search for and prefer hosts that would most favor their offspring’s or their own performances, respectively. Thus, we expected that the ovipositional preference of females would positively align with the performance of their larvae, including how larval performance may be mediated by expected (future) and ongoing (present) herbivory. Also, we expected that neonate larvae would colonize hosts randomly, given their dispersal on wind currents (ballooning), but would further disperse if they initially colonized poor hosts, while older (3^rd^-instar) larvae would orient towards and prefer hosts that would most favor their performance. Throughout, we assumed that plant-insect chemical communication would be paramount in mediating the host preferences and searching behaviors of both fall armyworm females and larvae. Also, we assumed that commercial-rearing did not alter the host preferences and searching behaviors of the moths and larvae used in our study, compared to field-collected fall armyworm individuals.

In reference to our expectations, first we found that fall armyworm females preferred the hosts on which their larvae performed poorest, opposite to what we expected. Also, and partially inconsistent with our expectation, we found that while the host preference of females was mediated by the occurrence of herbivory, it was ultimately mediated by the timing of herbivory: Females favored hosts on which herbivory was expected (i.e. bearing newly-laid, conspecific eggs), and averted hosts on which herbivory was ongoing (i.e. bearing conspecific larvae). Finally, our results concerning host finding and preferences of larvae were inconsistent with our expectations. In particular, on one hand, our results suggested that neonate larvae may colonize and settle on hosts according to their potential performance on a given host, rather than randomly as we expected, and on the other hand, they suggested that older (3^rd^-instar) larvae may search randomly for suitable hosts, seemingly guided by visual cues, opposite to what we expected.

### Host preference *vis-à-vis* offspring performance

We expected our results for this portion of the study to align with the prediction of the preference-performance hypothesis that females would preferentially oviposit on hosts on which their offspring’s performance is enhanced [2,43]. However, females laid the most eggs on the host on which their larvae’s performances would be poorest, maize line Mp708, and the fewest eggs on the host on which their larvae’s performances would be best, B73-*lox10*. While our results were clearly inconsistent with our expectations, they highlighted how preference-performance relationships may be mediated by a variety of ecological and life-history variables beyond offspring performance [2,43,62].

An obvious difference between the least (B73-*lox10*) and most (Mp708) preferred hosts is that the former, unlike the latter, is deficient in JA, GLV, and terpene production, which is relevant to herbivory by leaf-chewing insects, such as fall armyworm [57]; however, Mp708, the most preferred host, is known to constitutively produce high levels of JA [58]. Notably, though, any differences in GLV and terpene production between those hosts are likely irrelevant because of the absence of herbivory in this experiment. Thus, the remaining, plausibly relevant difference between the least- and most preferred hosts in our experiment may be their constitutive levels of JA, but its relevance, if any, remains to be tested.

While herbivore-induced JA, GLV, and terpene production seemed irrelevant to our results, two, non-mutually exclusive, ecological variables may explain in part the evident mismatch between female choice and larva performance. The first variable concerns dispersal of neonate larvae away from the host on which they hatch, which may override any host preference exhibited by females. Prior studies suggested that female preference for high-quality hosts for larvae may be more evident where larval dispersal is limited, which is not the case for fall armyworm in which neonate larvae disperse by ballooning [1,12,43,62,63]. Indeed, our experiment on host finding by neonates (*Host finding… neonate larvae*) revealed that a large majority of larvae dispersed from the plant on which they hatched, and most did so within 24 h of hatching (data not shown). Moreover, dispersal away from hosts bearing numerous larvae is likely adaptive because it would reduce intra-specific competition and cannibalism, the latter of which occurs beginning at early age (2–4 d-old larvae) in fall armyworm, and intensifies in later age (10–12 d-old) and as food dwindles [64]. Additionally, prior studies showed that fall armyworm’s host range exceeds 180 species [11,19–23], which enhances the likelihood that dispersing neonate larvae will colonize suitable, alternate hosts, as documented in early studies [12]. Thus, the propensity for dispersal of neonate larvae may override the moth’s host choices and be adaptive, while female ovipositional preferences may be dynamic, i.e. shaped by variables other than their offspring’s performance, such as risk of parasitism or egg mortality, which may be high and differ among hosts [2,65,66]. For example, recent field studies showed that fall armyworm egg-stage mortality on maize was 73 to 81% [16], and that parasitism and predation risks of fall armyworm larvae were three- to four-fold higher on the maize wild ancestor Balsas teosinte (*Zea mays parviglumis*) compared to maize [33].

A second ecological variable, herbivore host range, may help explain the evident mismatch between fall armyworm female choice and larva performance. A recent meta-analysis indicated that a close association between host preference and offspring performance is more likely in species with narrow versus broad host ranges [43], while the host range of fall armyworm exceeds 180 plant species from 42 families, as noted above. Additionally, earlier studies indicated that, compared to specialist species, those with broad host ranges may have sensory limitations that constrain their ability to discriminate between high- and low-quality hosts [67]. Such a constraint, along with ecological and life-history variables, may help explain why in some contexts, but not others, fall armyworm females discriminate between seemingly good and comparatively poor hosts. For example, in this study females discriminated between healthy plants and plants suffering herbivory (see Fig 3A), but not between highly resistant Mp708 plants and modestly resistant B73 plants (see Fig 1B and 1C); similarly, in other studies, females did not discriminate between seemingly poor hosts, on which larvae performed poorly and suffered high parasitism and predation rates, and comparatively good hosts, on which larvae performed well and suffered low parasitism and predation rates [32,33].

### Adult host preference *vis-à-vis* herbivory

Our results showed that, independently of whether or not plants were deficient in JA, GLV, and terpene production, fall armyworm females more frequently oviposited on plants in which herbivory was expected compared to plants in which herbivory was not expected. Contrastingly, our results also showed that females less frequently oviposited on plants with ongoing herbivory compared to plants free of herbivory, and also less frequently on plants deficient in JA, GLV, and terpene production (B73-*lox10*) compared to non-deficient plants (B73), independently of herbivory. Put together, these results suggested that on one hand, the timing of herbivory, whether future or present, differently mediates ovipositional preferences, and on the other hand, that fall armyworm oviposition and herbivory differently affect any JA-, GLV-, or terpene-dependent maize signaling relevant to ovipositing females.

We expected that females would be averse to ovipositing on plants with prior oviposition because prior oviposition implies exposing offspring to stronger intraspecific competition, greater risks of cannibalism and parasitism, and primed and active plant defenses [1,57,68–70]. Though our finding was contrary to our expectation, it seemed consistent with the results of a recent study suggesting that female’s may benefit from ovipositing on plants with prior oviposition. That study showed that by inducing salicylic acid (SA)-dependent defenses, oviposition suppressed oxylipin-mediated defenses controlled by the JA-pathway, and enhanced the performance of subsequent larvae [51]. However, that study showed enhanced larval performance only when the prior oviposition and subsequent larvae were of different species, but not when they were conspecific. Similarly, another study showed that the performance of fall armyworm larvae on maize did not appear to be affected by prior, conspecific oviposition [53]. Thus, it seems that prior conspecific oviposition would not benefit fall armyworm females through enhanced performance of their larvae. Nonetheless, our results clearly showed that females were able to discriminate between plants with or without prior oviposition, yet favored the former (see Fig 2). The females in our study may have responded to either increased SA- or decreased JA-levels in plants with prior oviposition, changes that were shown to be associated with prior oviposition [51]. Importantly, though, our results do not support a role for decreased JA-levels in preference for plants with prior oviposition because in our experiment oviposition frequencies were unaffected by whether plants were JA-deficient (B73-*lox10*) or not (B73). Thus, female preference for plants with prior oviposition may be linked to an oviposition-induced increase in SA levels. A prior study suggested that independently of whether oviposition affects direct plant defenses against fall armyworm, it may affect plant-insect interactions broadly [53]. That study showed, for example, that while larvae did not gain a performance advantage on maize plants with prior oviposition, oviposition suppressed the emission of herbivore-induced volatiles, which are used as host-location cues by parasitoids and predators, as well as emission of linalool, which is attractive to late-instar larvae. Another study, involving *Trichoplusia ni* (Hübner) and soybean, also showed that oviposition suppressed linalool emission [71]. Thus, fall armyworm females may benefit from ovipositing on plants with prior oviposition by reducing their larvae’s mortality risks from parasitism and cannibalism, rather than by enhancing their performance. A different study suggested that preference for plants with prior oviposition may indicate conspecific attraction in which females would benefit from preferring host plants previously selected by conspecifics [72]. It is unclear which, if any, of the benefits ascribed to conspecific attraction may apply to fall armyworm, whether time saved in finding high-quality hosts plants, predator satiation, group defense against natural enemies, decreased per capita risk of parasitism, or enhanced foraging efficiency and thermoregulation [72]. While multiple ecological variables may help explain fall armyworm’s preference for plants with prior oviposition, it is worth highlighting that oviposition-induced suppression of linalool emission [53,71] is expected to camouflage plants so that they are less apparent to ovipositing females, an expectation that in light of our results warrants further examination.

Similar to our experiment involving plants with prior oviposition, we expected that females would be averse to ovipositing on plants with ongoing herbivory, where their larvae would be exposed to stronger intraspecific competition, greater risks of cannibalism and parasitism, and primed and active plant defenses [1,57,68–70]. In this case, our results were consistent with our expectations. However, in contrast to our results with plants with prior oviposition, plant type mediated female preference under ongoing herbivory: females laid fewer eggs on plants deficient in JA, GLV and terpene production (B73-*lox10*) compared to non-deficient plants (B73), a finding that was surprising for at least two reasons. Firstly, because larval performance is enhanced on JA-deficient plants, which are poorly defended against fall armyworm (see Fig 1A), and secondly because females lay eggs during night-time, when maize plants (both JA and GLV-deficient and non-deficient) do not emit herbivore-induced plant volatiles [57]. Interestingly, an early study showed that (i) plants suffering herbivory release herbivore-induced plant volatiles during both day and night, (ii) the released volatiles differed between day and night, and (iii) night-active moths, such as fall armyworm, use night-time volatile blends to select (or reject) hosts for oviposition [73]. However, a recent study comparing the JA and GLV-deficient and non-deficient plants used in this study suggested that emission of herbivore-induced plant volatiles is under circadian control, with no volatiles released during the night [57]. Therefore, the herbivore-induced plant volatiles released by JA, GLV, and terpene-deficient and non-deficient plants may not differ during the night, and night-active females may have been incapable of discriminating between those plants, unless other cues are relevant or future studies show that maize plants release herbivore-induced plant volatiles during the night. Overall, while this experiment’s results are consistent with ecological hypotheses explaining why less oviposition is expected on plants with ongoing herbivory (e.g., increased parasitism risk, enhanced plant defenses), they seem inconsistent with predictions based on plant defense biochemistry and plant-herbivore communication. Particularly, further variables in addition to emissions of herbivore-induced plant volatiles or circadian control of herbivore-induced plant volatiles must mediate the host preferences of fall armyworm females.

### Host finding and preference of offspring

Within any constraints imposed by their limited mobility relative to females, we expected that larvae would search for and prefer hosts that would most favor their own performances. Thus, while we expected that neonate larvae would initially colonize hosts randomly, given their dispersal by ballooning, we expected that when they colonized a poor host they would disperse further to settle on hosts that favor their performance. For older larvae, we expected that they would orient to and prefer hosts favoring their performance, given their comparatively greater, directed mobility. However, our results showed on one hand that neonate larvae did not colonize and settle on hosts at random. On the other hand, our results showed that older larvae foraged randomly, seemingly independently of any volatile cues and dependent on visual cues, and were not discriminative upon encountering a host (Figs 5 and 6).

Neonate lepidopteran larvae disperse from their natal plants for a variety of reasons, from avoiding predation and competition to finding suitable hosts [1,74]. A survey addressing dispersal in neonate larvae of Lepidoptera suggests that in gregarious species (i.e. in which females lay eggs in masses), particularly in species that disperse via ballooning: (i) most neonate larvae from a single brood disperse within the first 24 h; (ii) older larvae infrequently disperse once they encounter a suitable host; (iii) larvae frequently respond to host plant volatiles, and; (iv) neonate larvae will more readily disperse away from poor hosts compared to suitable hosts, indicating that host quality may mediate their dispersal from natal hosts [1,75–79]. While our greenhouse experiments did not address how neonate and older larvae search for suitable hosts, prior laboratory studies showed that neonate and older larvae may use plant volatiles to orient to host plants [47,80–82]. For example, in a recent study neonate fall armyworm larvae on cowpea [*Vigna unguiculata* (L.)] seedlings were arrested (i.e. did not disperse) more frequently on seedlings with “old” feeding injury (4 h) caused by conspecific larvae compared to uninjured seedlings or seedlings with “new” injury (1 h); also, neonate larvae responded similarly in olfactometer assays by crawling more frequently towards volatiles from seedlings with old injury compared to seedlings without or with new injury [81]. Interestingly, the volatiles found to be attractive to neonate larvae in that study appeared to have conflicting functions, serving on one hand to orient neonate larvae, and on the other hand orient natural enemies to herbivore-damaged hosts. Our experiment with neonate larvae, in contrast, addressed dispersal away from the natal host and via ballooning, so presumed that dispersal would be mediated by wind currents, and colonization would be independent of any volatiles emitted by plants. However, we found that colonization was not random, which suggests that neonate larvae may direct to some degree their descent upon plants.

Directed aerial descent has been documented in wingless arthropods, including ants, jumping bristletails, and spiders, and seemed to rely on visual cues [83–85]; similarly, lepidopteran larvae have been documented parachuting to the ground on leaves of their hosts [86,87]. In every case, the ability to direct or slow descents seemed to be adaptive: Ants, jumping bristletails, and spiders directed their descents towards their host tree’s trunk, so facilitating a return to nests, suitable hosts, or familiar territory, and; lepidopteran larvae slowed their fall to the ground prior to pupation or changed the feeding venue from plant canopy to soil leaf litter, and perhaps minimized their exposure to induced plant defenses. A directed descent upon suitable host plants is plausible and would similarly be adaptive in neonate fall armyworm larvae given that they were shown to orient towards host plant volatiles, and they suffer high egg (71–81%) and early-instar larval (> 95%) mortality in maize fields, as they did in our greenhouse trials in which the larvae that settled on host plants amounted to < 2% of the deployed eggs [16,81]; other studies involving neonate or older lepidopteran larvae also showed the high mortality rates for larvae crawling on soil to find hosts [1,74]. While our study did not specifically address directed aerial descent in neonate fall armyworm larvae, our results indicate that a close examination of such behavior is warranted.

Our study did not address the possibility that neonate fall armyworm larvae use leaf surface features for selecting suitable hosts. Neonate larvae may use leaf hairs, trichomes, and waxes, and a variety of leaf surface compounds to evaluate the suitability of a host plant [1]. Thus, neonate fall armyworm larvae fed more, grew larger, and traveled greater distances on leaves of a resistant maize genotype when the abaxial surface’s cuticular lipids were extracted, compared to leaves with intact cuticular lipids [28,29,88].

In the case of older larvae, we observed that they made rhythmic side-to-side head movements (“wig-wagging”) while foraging in the experimental arenas, a behavior that in a prior study was associated with transverse klinotaxis, an odor-induced movement [89]. However, other studies suggested that this behavior enhances the spatial vision of larvae when a target is within the visual field [90]. Fall armyworm larvae displayed this behavior upon approaching maize plants, but also when approaching model plants, suggesting that wig-wagging may serve to enhance their spatial vision rather than their perception of volatiles. Indeed, we found that fall armyworm larvae did not show any preference for real versus model maize plants, suggesting that plant volatile compounds were irrelevant to host searching, consistent with observations that the chemosensory apparatus of larval lepidoptera appears to be limited in comparison with that of corresponding adults. For example, while *Spodoptera littoralis* (Boisduval) larvae express 22 olfactory receptors, adults express 47 receptors [91]. Concordantly, a recent study suggested that *S*. *littoralis* larvae respond to a broad range of odors, rather than specifically to select odors [92]. Moreover, older fall armyworm larvae search for hosts by crawling on the soil surface, where the value of volatiles as cues is uncertain. While the visual perception of fall armyworm adults has been studied [93], similar studies are not available for their larvae nor of larvae of other lepidopteran species, which lack compound eyes. Overall, however, it is possible that fall armyworm larvae use both visual and chemical cues for finding suitable host plants, similar to other insects.

### Ecology of host selection and implications for fall armyworm management

Our results are relevant in two intertwined contexts. In an ecological context, they add to our understanding of host selection in herbivorous insects of three functionally distinct stages (neonate and older larvae, and adults), as mediated by host plant quality and conspecific herbivory. In an agricultural context, our results provide insight for developing pest management strategies for scenarios in which crop hosts vary in quality, whether at the landscape level (among fields) or at the field level (among plants), or both.

**Fig 7:**
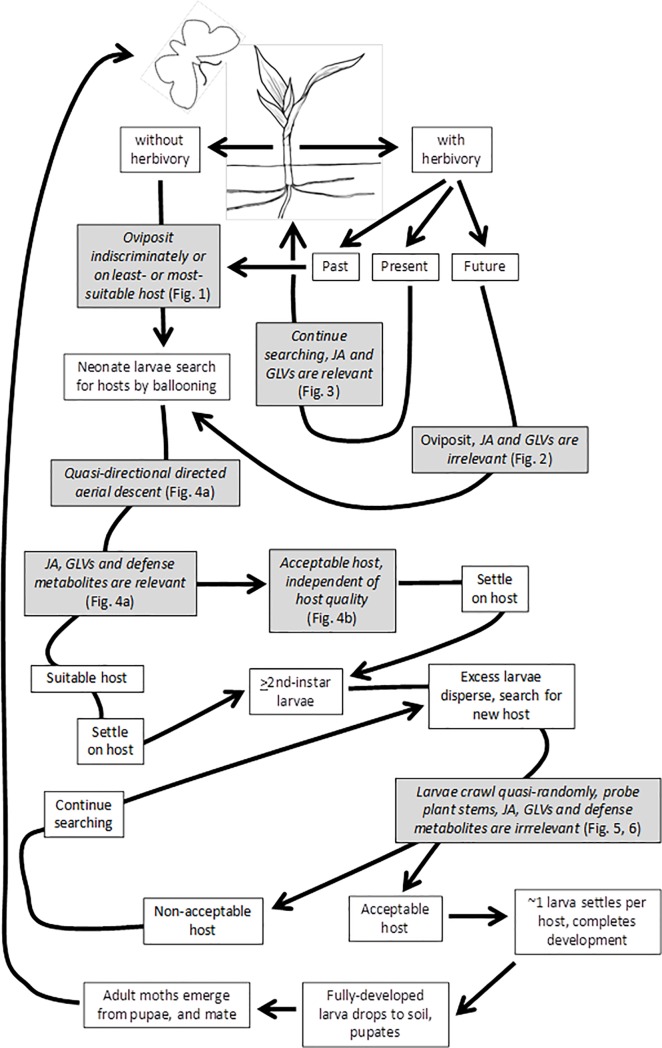
Hypothetical model for host searching and selection processes of fall armyworm females and larvae (neonate and older), based on literature sources and this study’s results (see Discussion for literature sources). This study’s findings are pertinent to various stages of the searching and selection processes, so were incorporated into the model as hypotheses for further study, and are indicated by filled boxes containing italicized text.

Per the available literature and our research, the host searching and selection processes of fall armyworm females and larvae on maize can be outlined as follows (Fig 7). Once mated, females search for host plants; plant volatiles may or may not be relevant to orientation, depending on multiple variables, including presence, absence, or expectation of herbivore injury; and, chemical and mechanical cues mediate oviposition on host plants [94]. Females oviposit 4–10 egg masses during their adult lives, and each mass may contain ~500–1,300 eggs; eggs hatch 3–4 d after oviposition at 25°C [95,96]. Upon hatching, most larvae leave natal host plants by ballooning, and may colonize more suitable plants; neonate larvae may be capable of quasi-directional directed aerial descent; older larvae disperse by crawling, and may search for hosts at random, relying on visual cues [12]. Larvae usually pass through six instars [97], but the actual number varies from 5 to 9, depending on temperature and diet [98,99]. Fully-developed larvae drop to the soil for pupation, and the pupal stage lasts ca. 9 d at 27°C [96].

In an ecological context, our results enrich our understanding of the host selection and finding processes of adult and larval herbivores (Fig 7). In the context of other studies, our results indicate that in the absence of herbivory, females may choose apparently poor hosts for their offspring, may be indiscriminant when selecting among conspecific host plants (and heterospecific host plants) (e.g., [19,20,33,100,101]), or may discriminate among host plants of different species or qualities (e.g., [27,101–103]). Importantly, some studies showing discrimination failed to account for potential or actual differences in plant sizes or leaf surface areas among the hosts that were compared, in addition to other experimental concerns. In contrast, females in our study discriminated on the basis of whether host plants are suffering or will suffer herbivory. Specifically, females favored plants hosting recently-laid eggs, i.e. in which conspecific herbivory was expected, over plants free of eggs, independently of the production of GLVs and terpenes by plants, and; they discriminated in favor of plants free of present, conspecific herbivory over plants presently suffering herbivory, and in favor of plants emitting induced GLVs and terpenes over plants deficient in GLV and terpene emissions. Preference for plants hosting eggs over plants free of eggs may be explained by multiple, sometimes conflicting, ecological variables, as noted above, so this finding merits further examination in order to better understand its underlying causes and any ecological implications. In contrast, the ovipositional preference for plants free of ongoing herbivory found in this study is consistent with the findings of other studies and with ecological predictions, though is seemingly inconsistent with predictions based on the biochemistry of plant-herbivore communication, as noted above.

Our results concerning dispersal and host finding and preferences of larvae suggested on one hand that neonate larvae may not disperse entirely at random, as presumed, and that older larvae rely on visual rather than olfactory cues when searching for hosts, so that host searching appears to be quasi-random in older larvae (Fig 7). Notably, our results for neonate larvae suggested that they may be capable of directed aerial descent, and showed that they more frequently colonize suitable over less-suitable plants, both findings being consistent with ecological predictions, especially in the context of high mortality rates of neonate larvae, as noted above. However, our observation concerning directed aerial descent merits further study because of its ecological implications, and because it has not been documented previously in lepidopteran larvae. Our findings with older larvae suggested that plant volatile compounds are irrelevant to host finding, and pointed to reliance instead on visual cues for finding hosts, though prior studies showed that larvae were attracted to plant volatiles, as noted above. Thus, this finding for older larvae also merits further examination.

In an agricultural context, our results provide insights for developing management strategies targeting fall armyworm in maize, particularly in the subtropical and tropical Americas and sub-Saharan Africa. Developing novel management strategies is important given that past efforts have been minimally successful in the Americas, and effective strategies are urgently needed in Africa, where fall armyworm is a devastating, invasive pest [15,17,18,104–106]. In a pest management context in particular, our results are especially relevant to developing or improving push-pull strategies against fall armyworm that exploit maize signaling associated with present and future herbivory, and GLV and terpene emissions. Push-pull pest management strategies have proven effective in various crops, including maize, and an effective approach involving trap crops against fall armyworm was reported recently [107–110]; parallel approaches, based on manipulation of herbivore-induced plant volatiles (HIPVs) have also been proposed [41]. In particular, a push-pull strategy against fall armyworm could be explored for its potential to “push” ovipositing females away from maize plants using volatiles that mimic those produced by plants undergoing herbivory, and to “pull” ovipositing females toward trap plants using volatiles that mimic those produced by maize plants hosting eggs. Moreover, the “push” component of such a strategy could be enhanced by integrating maize cultivars with low or nil GLV or terpene emissions. Also, a push-pull strategy involving trap plants likely would also benefit from the seemingly random manner in which older larvae search for host plants because trap plants would likely intercept a fraction of the foraging larvae, independently of the plant’s association with fall armyworm. Finally, a push-pull strategy targeting fall armyworm could be further improved by incorporating host plant resistance in the form of fall armyworm-resistant maize cultivars, which likely would be colonized less frequently by ballooning, neonate larvae, and on which any colonizing larvae would perform poorly. Overall, our results point to the value of further considering “agroecological” approaches that seek to manipulate or interfere with herbivore host finding and selection—such as intercropping, weed management, and trap cropping—, for fall armyworm management in the tropical and subtropical Americas and sub-Saharan Africa, as argued previously for a variety of maize pests [111–115].
